# Differences in Symptom Burden in Primary Brain Tumor Patients Based on Sex, Race, and Ethnicity: a Single-Center Retrospective Study

**DOI:** 10.1007/s40615-023-01761-9

**Published:** 2023-10-02

**Authors:** Kendall Brady, Adam L. Cohen

**Affiliations:** 1 National Cathedral School, Woodley Road NW, 20016 Washington, DC, USA; 2 Inova Schar Cancer Institute, 8081 Innovation Park Dr., VA 22031 Fairfax, USA

**Keywords:** Brain tumor, Glioma, Symptom burden, Sex factors, Race factors

## Abstract

**Background:**

Symptom burden affects quality of life and prognosis in primary brain tumor (PBT) patients. Knowing whether symptom burden varies based on sex, race, or ethnicity may affect the interpretation of the relationship between symptoms and survival may reveal issues with applying the tools to measure symptom burden to different groups and may identify inequities in symptom management that need to be addressed at a system level. To determine whether symptoms in PBT patients vary across demographic groups, we conducted a retrospective chart review of symptom burden collected as part of routine care in a diverse population.

**Methods:**

Patient demographics and scores on the MD Anderson Symptom Inventory-Brain Tumor (MDASI-BT) module were extracted from the electronic medical record for patients seen in the Inova Neuro-oncology Clinic between March 2021 and June 2022. MDASI-BT scores were compared based on side of tumor, sex, race, and ethnicity for the entire population and for the subset with gliomas.

**Results:**

We included 125 people, of whom 85 had gliomas. For both the entire group and the subgroup with gliomas, about 40% were female and about 40% were non-White race. No differences in symptom burden were seen between males and females. Pain and numbness/tingling symptom burden were higher in both the entire population and the glioma subgroup for people of Hispanic/Latino/Spanish ethnicity and for people of races other than White or Middle Eastern self-identification.

**Conclusions:**

Pain, weakness, and numbness/tingling varied significantly across racial and ethnic groups. Further research is needed to validate this finding in other populations and determine its cause.

## Introduction

Over 80,000 people in the USA each year are diagnosed with a primary brain tumor (PBT) [1]. Gliomas are the most common malignant PBT among adults [2]. Though treatment options for PBTs have been thoroughly studied, quality of life for both patients and caregivers is increasingly the subject of neuro-oncology research [2]. Research on patient quality of life, often focused on symptom burden, aims to better support patients and families within the healthcare system and alleviate patients’ distress [3].

PBT patients may experience a variety of symptoms, with seizures, cognitive deficits, and drowsiness being the most common symptoms across the disease trajectory among glioma patients [4]. Symptom burden is important to study both because of its importance for the patient experience but also because it correlates with quality of life, recurrence, and survival [2, 5, 6]. Numerous studies investigate the impacts of race, ethnicity, and sex on the experience of brain tumor patients. These studies have focused primarily on issues of access, survival, and tumor biology [7–12]. To our knowledge, no studies investigate the prevalence of PBT tumor symptoms across multiple demographic groups defined by race, ethnicity, age, and sex.

For PBT patients, the MD Anderson Symptom Inventory-Brain Tumor (MDASI-BT) module is a validated indicator of patients’ symptom burden [5]. However, most studies of quality of life and symptom burden in PBT have either not assessed the sex, race, or ethnicity of the study populations or included mostly White, non-Hispanic, and male participants. More research including diverse groups is needed to produce the most accurate and representational descriptions of the patient experience.

This study aimed to describe the symptoms and symptom burden of PBT patients, and glioma patients in particular, stratified by demographic factors through a retrospective chart review of MDASI-BT data. By investigating the intersection of demography and symptom burden, providers can formulate more effective interventions to alleviate individual’s symptoms, including developing treatments that target specific symptoms, and identify systemic inequity in care.

## Methods

### MDASI-BT

We employed a data set originated from the MDASI-BT module, a validated self-administered questionnaire that records symptom burden and interference on a 1–10 scale [5]. All patients in the Inova Neuro-oncology Clinic were asked to completed the MDASI-BT at first visit and every 3 months starting in March 2021 (Licensed March 10, 2021, in English, Spanish, and Chinese). The MDASI-BT has 22 items for symptom burden and 6 items for symptom interference. The items for symptom burden and for symptom interference are then averaged for the two subscores. We report averages for each individual item and for the subscores.

### Study Design and Participants

Participants were included if they were 18 or over. Data was extracted by manual review of the electronic medical record (EMR, Epic) from visits to the neuro-oncology clinic that occurred between February 1, 2021, and July 7, 2022. Data was extracted by one author with spot verification by the other author. Age, race, ethnicity, and legal sex were self-reported in the EMR. The following races were available in the medical record: Another Race, Asian, Black or African American (Black), Hispanic or Latino (Latino), Middle Eastern, Unavailable, and White or Caucasian (White). Our patients identified as the following ethnicities available to self-select in the medical record: Hispanic/Latino/Spanish origin (Latino), not Hispanic/Latino/Spanish origin (not Latino), and Unavailable. One patient used free text to identify their ethnicity as American. For each patient, we recorded the tumor type; tumor grade based on World Health Organization classification from 1 (more benign) to 4 (more malignant); tumor location; tumor side; age at diagnosis; tumor recurrence; race; ethnic group; reported sex; date of diagnosis; whether MDASI-BT was completed at diagnosis; date of MDASI-BT; MDASI-BT scores at diagnosis, including severity and interference scores; MDASI-BT scores at other times; Karnofsky performance status (KPS) at the time of MDASI-BT; and survival. KPS, which is a clinician-determined assessment of functional capacity that ranges from 0 (death) to 100 (normal, no complaints, no evidence of disease), for which a score of 70 represents the ability to do activities of daily living, was listed in notes by the treating neuro-oncologist as part of clinical care [13].

### Statistical Analysis

Statistical analysis was completed using Stata/BE 17.0. Participant demographics were summarized using medians, means, ranges, and percent. Relationships between demographic variables were assessed using chi-square likelihood ratio tests for categorical variables and *t* test and ANOVA for continuous variables. The overall symptom burden scores and subscores were summarized using box and whisker plots with means and interquartile ranges reported and compared between groups using *t* test and ANOVA. Analyses were done using all available MD Anderson Symptom Inventory (MDASI) results. Results from limiting to just the first MDASI for each person were similar.

### Privacy

All data and records generated during this study were kept confidential in accordance with Inova Health System institutional policies and HIPAA on participant privacy.

### Ethics Statement

A waiver of consent was obtained from the IRB. The study was approved by the Inova IRB.

## Results

We included 125 people, of whom 85 had gliomas (Table 1). The majority of our population self-identified as male (whole population: 58% male, gliomas: 60% male), mirroring the small male predominance among national cohorts [1]. Glioblastomas were the most common tumor (whole group: 34% of cohort, gliomas: 51%), as expected. In the glioma group, the tumors were most commonly located in the frontal and temporal lobes (frontal: 43%, temporal: 21%). The mean age at diagnosis was 53.3 for the whole group and 51.9 for the subgroup with gliomas (Table 2); however, Latino patients had a significantly lower mean age than the rest of the cohort, whether looking at those who self-identified as Latino as their race (*p* = 0.0013) or their ethnicity (*p* = 0.0008). In the entire group, the mean age of people who identified their ethnicity as Latino was 41 years compared to 56.5 years for people who identified as not Latino. The same pattern was held for patients with glioma (Table 3). The difference in age between Latino and non-Latino groups is similar to that reported in the national PBT population [14].

**Table 1:** Study population and demographics

	Grand total whole group, *N* = 125			Grand total gliomas, *N* = 85	
	*n*	%		*n*	%
Legal sex					
F	52	41.60		34	40.00
M	73	58.40		51	60.00
Race					
Another Race	17	13.60		14	16.47
Asian	11	8.80		4	4.71
Black or African American	9	7.20		6	7.06
Hispanic or Latino	6	4.80		5	5.88
Middle Eastern	1	0.80		1	1.18
Unavailable	8	6.40		6	7.06
White	73	58.40		49	57.65
Ethnicity					
American	1	0.80			
Hispanic/Latino/Spanish origin	20	16.00		15	17.65
Not of Hispanic/Latino/Spanish origin	93	74.40		60	70.59
Unavailable	11	8.80		10	11.76
Grade					
1	14	11.20		3	1.08
2	28	22.40		19	13.67
3	19	15.20		15	16.19
4	48	38.40		48	69.06
No grade	16	12.80			
Diagnosis					
Astrocytoma	18	14.40		17	20.00
CNS lymphoma	4	3.20			
Craniopharyngioma	1	0.80			
Diffuse midline glioma	3	2.40		3	3.53
Ependymoma	7	5.60		7	8.24
Ganglioneuroblastoma	1	0.80			
Germinoma of CNS origin	1	0.80			
Glioblastoma	43	34.40		43	50.59
Hemangioblastoma	2	1.60			
Intracranial nongerminomatous germ cell tumor	1	0.80			
Meningioma	20	16.00			
Oligodendroglioma	14	11.20		14	16.47
Paraganglioma	1	0.80			
Pineal parenchymal tumor of intermediate differentiation	1	0.80			
Pineocytoma	1	0.80			
Schwannoma	1	0.80			
Solitary fibrous tumor of the CNS	1	0.80			
Unbiopsied tumor	4	3.20			
Xanthoastrocytoma	1	0.80		1	1.18
Side					
Both	7	6.42		2	2.60
L	57	52.29		38	49.35
R	45	41.28		37	48.05
Location					
Brain stem	1	0.72			
Cerebellopontine angle	3	2.16		2	2.20
Cerebellum	3	2.16		1	1.10
Cervicomedullary junction	1	0.72		1	1.10
Corpus callosum	4	2.88		4	4.40
Frontal lobe	53	38.13		39	42.86
Insula	2	1.44		2	2.20
Multifocal	1	0.72			
Nasal sinuses	1	0.72			
Occipital lobe	10	7.19		5	5.49
Orbits	1	0.72			
Parietal lobe	12	8.63		9	9.89
Pineal gland	3	2.16			
Skull base	5	3.60			
Spine	8	5.76		5	5.49
Temporal lobe	23	16.55		19	20.88
Thalamus	6	4.32		4	4.40
Ventricle	2	1.44			
Recurrence					
No recurrence	81	64.80		51	60.00
Recurrence	44	52.20		34	40.00
Survival					
Alive	114	91.20		75	88.24
Deceased	11	8.80		10	11.76
MDASI at diagnosis					
No	94	75.20		61	71.76
Yes	31	24.80		24	28.24
KPS at the time of diagnosis					
≥ 70	120	97.48		82	97.94
< 70	5	2.52		3	2.06

The characteristics of the entire population and of the people with gliomas included in the study are shown. Percent may not add up to 100% due to rounding and because some patients could have tumors in multiple locations. Race and ethnicity are reported exactly as they are listed in the electronic medical record

Abbreviations: *M* male, *F* female, *L* left, *R* right

**Table 2:** Age and time to diagnosis in the whole group and glioma group

	Mean	Median	Minimum	Maximum	25th percentile	75th percentile
Whole group						
Age at diagnosis (years)	53.3	55	1	89	42	67
Time from diagnosis to first MDASI (days)	995	55	5	8571	107	862
Gliomas						
Age at diagnosis (years)	51.8	53	1	89	41	64
Time from diagnosis to first MDASI (days)	833	230	5	8571	100	569

**Table 3:** Relationship between clinical and demographic factors

	Sex, *n* (%)		Race, *n* (%)						Ethnicity, *n* (%)		
	M	F	Another Race	Asian	Black	Latino	Unavailable	White	Latino	Not Latino	Unavailable
Total	51	34	14	4	6	5	6	49	15	60	10
Diagnosis											
Astrocytoma	13 (25)	4 (12)	3 (21)	0 (0)	2 (33)	0 (0)	1 (17)	11 (22)	3 (20)	13 (22)	1 (10)
Diffuse midline glioma	2 (4)	1 (3)	1 (7)	0 (0)	0 (0)	1 (20)	0 (0)	1 (2)	1 (7)	1 (2)	1 (10)
Ependymoma	4 (8)	3 (9)	3 (21)	0 (0)	1 (17)	1 (20)	1 (17)	1 (2)	4 (27)	1 (2)	2 (20)
Glioblastoma	23 (45)	20 (59)	2 (14)	4 (100)	2 (33)	2 (40)	3 (50)	30 (61)	3 (20)	35 (58)	5 (50)
Oligodendroglioma	8 (16)	6 (18)	4 (29)	0 (0)	1 (17)	1 (20)	1 (17)	6 (12)	3 (20)	10 (17)	1 (10)
Xanthoastrocytoma	1 (2)	0 (0)	1 (7)	0 (0)	0 (0)	0 (0)	0 (0)	0 (0)	1 (7)	0 (0)	0 (0)
*p*	0.69		0.29						0.02		
Location											
Cerebellopontine angle	2 (4)	0 (0)	0 (0)	0 (0)	0 (0)	0 (0)	0 (0)	2 (4)	0 (0)	1 (2)	1 (10)
Cerebellum	0 (0)	1 (3)	0 (0)	0 (0)	0 (0)	1 (20)	0 (0)	0 (0)	1 (7)	0 (0)	0 (0)
Cervicomedullary junction	1 (2)	0 (0)	0 (0)	0 (0)	0 (0)	0 (0)	1 (17)	0 (0)	0 (0)	0 (0)	1 (10)
Corpus callosum	2 (4)	2 (6)	1 (7)	0 (0)	0 (0)	1 (20)	0 (0)	2 (4)	1 (7)	2 (3)	1 (10)
Frontal lobe	24 (47)	15 (44)	7 (50)	2 (50)	3 (50)	2 (40)	2 (33)	22 (45)	6 (40)	29 (48)	4 (40)
Insula	1 (2)	1 (3)	0 (0)	0 (0)	0 (0)	0 (0)	1 (17)	1 (2)	0 (0)	1 (2)	1 (10)
Occipital lobe	3 (6)	2 (6)	0 (0)	0 (0)	0 (0)	0 (0)	0 (0)	5 (10)	0 (0)	4 (7)	1 (10)
Parietal lobe	3 (6)	6 (18)	2 (14)	1 (25)	0 (0)	0 (0)	2 (33)	4 (8)	2 (13)	5 (8)	2 (20)
Spine	2 (4)	3 (9)	3 (21)	0 (0)	1 (17)	0 (0)	0 (0)	1 (2)	3 (20)	2 (3)	0 (0)
Temporal lobe	11 (22)	8 (24)	0 (0)	2 (50)	2 (33)	1 (20)	0 (0)	14 (29)	1 (7)	18 (30)	0 (0)
Thalamus	4 (8)	0 (0)	1 (7)	0 (0)	0 (0)	0 (0)	0 (0)	3 (6)	1 (7)	3 (5)	0 (0)
*p*	0.18		0.76						0.03		
Age (mean)	52.5	49.9	42.7	61.3	51.3	35.6	52.5	55.5	40.5	54.4	53.5
*p*	0.65		0.041						0.009		

Distribution of diagnosis, location, and age among glioma patients is shown. Column percentages are listed. *p* values are reported for each box calculated by chi-square for categorical variables and ANOVA or *t* test for age. The Ethnicity category for “American” was omitted because there was only one person, and this is not a commonly accepted ethnic description

Our cohort included a majority of people who identified as White (whole group: 58%, gliomas: 58%) among races and as not Latino among ethnicities (whole group: 74%, gliomas: 71%), while non-White populations collectively made up 42% of the entire PBT cohort (Another Race: 14%, Asian: 9%, Black: 7%, Latino: 5%, Middle Eastern: 10%, Unavailable: 6%). Because our EMR included Hispanic or Latino as both a race and ethnicity option, we looked at how the two related. Of the 20 people who reported their ethnicity as Hispanic or Latino, 5 reported their race as Hispanic or Latino, 14 reported their race as Another Race, and 1 as White. No one reported their race as Hispanic or Latino and their ethnicity as Not Hispanic or Latino, but 1 person who reported their race as Hispanic or Latino did not report an ethnicity. There were no differences in tumor side, location, histology, or grade based on patient sex in either the entire population or the glioma subgroup (Table 3). There were small differences in tumor location based on race and ethnicity, with fewer temporal lobe tumors among the whole cohort in people who identified as Another Race or Latino. People of Latino ethnicity were more likely to have ependymoma and less likely to have glioblastoma than people who identified as Latino ethnicity (*p* = 0.023).

**Fig. 1:**
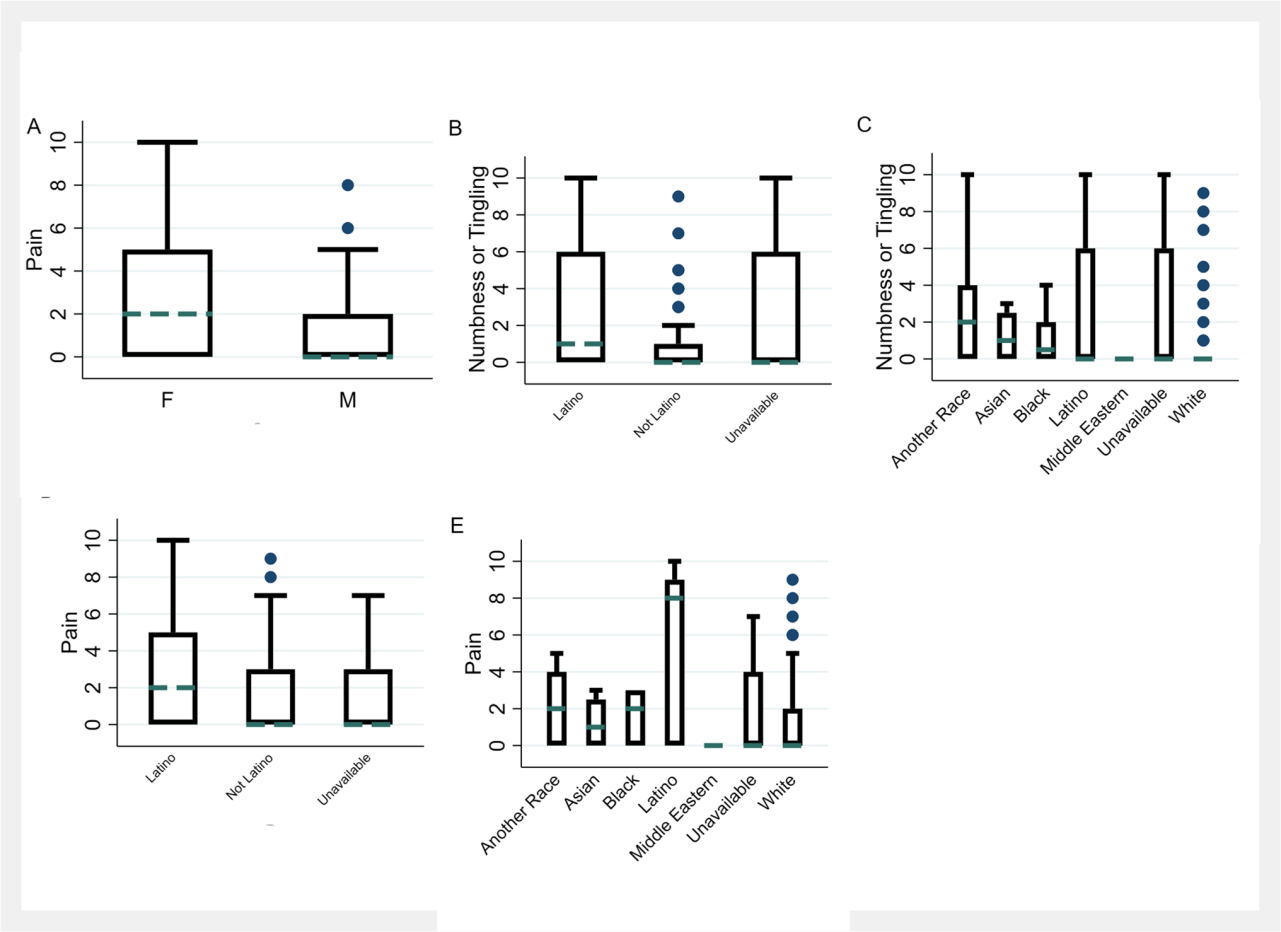
Box and whisker plots of selected MDASI results for pain (**A**, **D**, and **E**) and numbness/tingling (**B**, **C**) by legal sex (**A**), race (**C**, **E**), and ethnicity (**B**, **D**). Boxes indicate the median, 25th percentile, and 75th percentile. Whiskers indicate the adjacent values. Dots indicate outliers

We found no difference between the symptom burden of males and females, both in individual item scores and in the component scores (Table 4). However, when limiting to people with gliomas, females reported higher pain burden scores than males (*p* = 0.007) (Fig. 1A). In people with gliomas, females had a median pain score of 2, with mean 2.7 and standard deviation 3.1, versus a median score of 0 for males, with mean 1.2 and standard deviation 1.9.

**Table 4:** MDASI results

Items	Legal sex			Race							Ethnicity			
	M	F	*p* value	Another Race	Asian	Black	Latino	Unavailable	White	*p* value	Latino	Not Latino	Unavailable	*p* value
***N***	73	52		17	11	9	6	8	73		20	93	11	
Whole group														
Pain	1.72	2.02	0.31	2.00	1.44	1.25	4.50	1.57	1.33	*0.00*	3.00	1.33	1.27	*0.00*
Fatigue	3.23	3.43	0.54	2.50	3.00	3.88	4.25	2.86	3.37	0.25	3.29	3.23	3.55	0.55
Nausea	0.61	0.78	0.42	0.58	0.78	0.38	1.00	0.00	0.43	0.24	0.79	0.45	0.18	0.10
Disturbed sleep	2.14	2.15	0.96	2.42	2.78	1.50	2.00	2.14	1.87	0.16	2.64	1.95	1.64	*0.01*
Distress	2.05	2.57	0.1	2.00	4.00	3.13	6.00	1.29	2.16	0.12	3.36	2.28	2.64	0.71
Shortness of breath	0.85	0.87	0.89	1.25	1.22	1.13	1.00	1.29	0.86	0.70	1.36	0.88	1.27	0.40
Memory	2.81	2.65	0.62	2.33	2.89	2.88	2.50	2.57	2.79	0.92	2.64	2.68	3.18	0.82
Appetite	1.39	1.36	0.90	1.92	2.22	0.50	2.00	0.00	1.14	0.05	2.21	1.17	0.45	0.34
Drowsiness	2.37	2.50	0.70	2.08	2.11	3.25	2.00	2.29	2.87	0.20	2.21	2.69	3.09	0.11
Dry mouth	1.35	1.68	0.23	1.33	3.00	1.50	2.25	0.71	1.32	0.40	1.79	1.45	1.27	0.87
Sadness	1.75	2.31	0.09	1.75	2.44	2.88	6.50	0.86	1.90	*0.01*	3.29	1.85	2.55	0.23
Vomiting	0.23	0.31	0.57	0.75	0.89	0.00	0.00	0.00	0.14	0.12	0.64	0.21	0.09	0.06
Numbness/tingling	1.48	1.49	0.98	2.83	1.56	2.75	2.75	2.29	1.05	*0.00*	3.00	1.24	2.18	*0.00*
Weakness	1.61	1.90	0.40	2.50	1.56	3.13	4.25	0.86	1.71	*0.03*	3.29	1.71	1.91	*0.01*
Understanding	1.72	1.49	0.45	0.83	2.44	3.50	4.75	2.00	1.08	*0.01*	2.07	1.49	1.45	0.17
Speaking	2.25	1.74	0.10	1.17	2.22	3.63	5.00	2.29	1.71	*0.01*	2.36	2.01	1.55	0.45
Seizures	0.44	0.29	0.43	0.58	0.00	1.38	2.50	0.00	0.30	*0.01*	1.21	0.37	0.09	0.13
Concentrating	2.03	1.46	0.05	1.17	2.00	3.50	3.75	1.29	1.75	0.05	2.00	1.92	1.45	0.64
Vision	1.71	1.38	0.26	2.92	2.44	2.25	2.50	2.00	1.44	0.53	3.14	1.64	1.64	0.07
Appearance	0.76	0.78	0.92	0.58	0.44	2.25	2.50	0.29	0.46	*0.04*	1.07	0.67	0.27	0.15
Bowel	1.14	1.19	0.87	2.00	0.11	1.00	1.25	0.00	1.35	*0.03*	1.93	1.14	0.64	*0.03*
Irritability	1.93	1.79	0.63	2.58	1.56	3.75	3.50	2.43	1.62	*0.00*	3.14	1.77	2.36	0.17
General activity	2.81	2.52	0.47	4.50	2.67	2.75	2.50	3.14	3.02	0.38	3.86	2.96	3.36	0.14
Mood	2.43	2.56	0.72	3.33	3.22	2.75	4.00	3.00	2.41	0.36	3.50	2.53	3.09	0.09
Work	2.83	2.74	0.83	4.75	3.22	5.38	2.75	3.71	2.95	0.32	4.14	3.24	3.73	0.29
Relations	1.79	1.82	0.93	2.83	1.67	2.63	2.25	1.86	1.63	0.38	2.50	1.83	1.55	0.49
Walking	2.50	2.45	0.91	3.67	2.11	3.88	2.25	2.71	2.59	0.19	3.07	2.64	3.27	0.18
Enjoyment	2.53	2.79	0.48	4.25	2.22	3.13	2.25	3.29	2.70	0.19	3.93	2.62	3.55	0.13
Subscores														
Severity score	1.62	1.66	0.94	1.73	1.87	2.24	3.03	1.32	1.43	*0.02*	2.29	1.55	1.29	*0.05*
Interference score	2.47	2.50	0.93	3.89	2.52	3.42	2.67	2.95	2.55	0.23	3.50	2.64	3.09	0.12
Gliomas														
Pain	1.70	2.35	0.07	1.78	1.67	1.67	6.00	1.83	1.36	*0.00*	3.4	1.41	1.4	*0.01*
Fatigue	3.22	3.85	0.11	2.22	3.33	3.67	5.67	3.33	3.82	0.11	3.6	3.57	3.9	0.59
Nausea	0.60	0.86	0.30	0.00	1.33	0.50	1.33	0.00	0.41	0.30	0.4	0.45	0.2	0.24
Disturbed sleep	2.26	2.35	0.79	1.89	4.33	1.67	2.00	2.50	2.14	0.32	2.3	2.24	1.8	*0.02*
Distress	2.16	2.85	0.08	1.11	3.67	3.50	4.67	1.33	2.52	0.16	2.3	2.43	2.8	0.86
Shortness of breath	0.93	0.95	0.94	0.78	2.00	1.17	1.33	1.50	1.09	0.56	1.1	1.10	1.4	0.42
Memory	2.74	2.83	0.80	1.67	2.67	2.67	2.67	3.00	2.86	0.99	2.2	2.63	3.5	0.62
Appetite	1.58	1.53	0.89	1.89	3.33	0.67	2.67	0.00	1.32	0.07	2.5	1.31	0.5	0.05
Drowsiness	2.28	2.81	0.16	1.67	1.00	2.00	2.00	2.67	3.27	*0.01*	1.9	2.80	3.4	0.08
Dry mouth	1.45	1.92	0.16	0.89	4.33	1.00	2.67	0.83	1.68	0.25	1.6	1.65	1.4	0.73
Sadness	1.94	2.55	0.12	1.00	3.67	2.83	5.33	1.00	2.27	0.07	2.4	2.10	2.8	0.56
Vomiting	0.19	0.24	0.69	0.11	0.67	0.00	0.00	0.00	0.20	0.33	0.1	0.20	0.1	0.59
Numbness/tingling	1.21	1.46	0.46	3.44	1.67	1.17	3.33	2.67	0.91	*0.00*	3.8	0.92	2.4	*0.00*
Weakness	1.52	1.84	0.41	2.22	3.00	1.17	5.67	1.00	1.82	*0.03*	3.6	1.61	2.1	*0.01*
Understanding	1.71	1.50	0.56	0.67	2.67	2.50	6.00	2.33	1.05	*0.01*	2.4	1.31	1.6	0.05
Speaking	2.34	1.80	0.13	0.78	3.67	2.00	5.00	2.67	1.84	0.15	2.1	2.04	1.7	0.34
Seizures	0.56	0.28	0.25	0.44	0.00	1.83	3.33	0.00	0.43	0.01	1.4	0.57	0.1	0.34
Concentrating	1.78	1.58	0.55	1.33	2.33	2.33	3.67	1.50	1.59	0.20	2.2	1.67	1.6	0.30
Vision	1.49	1.22	0.38	2.33	1.33	0.50	3.33	2.33	1.05	0.09	3	0.98	1.8	*0.01*
Appearance	0.79	0.93	0.60	0.67	0.67	1.33	3.33	0.33	0.59	0.17	1.4	0.73	0.3	0.04
Bowel	1.12	1.29	0.54	1.89	0.33	0.83	1.67	0.00	1.50	0.15	2	1.31	0.7	0.09
Irritability	2.01	1.84	0.62	1.56	2.00	2.50	3.33	2.83	1.95	0.24	2.3	1.94	2.6	0.68
General activity	2.66	2.89	0.61	4.56	2.00	2.50	3.33	3.67	3.00	0.13	4.1	2.90	3.7	*0.05*
Mood	2.39	2.76	0.34	2.33	2.67	2.50	3.00	3.33	2.75	0.53	2.3	2.71	3.3	0.39
Work	2.75	3.11	0.44	5.22	2.00	4.67	3.00	4.00	3.07	0.28	4.6	3.22	3.9	0.18
Relations	1.75	1.90	0.68	1.56	1.67	1.67	3.00	2.00	1.82	0.76	1.5	1.94	1.6	0.84
Walking	2.08	2.66	0.19	3.22	0.67	2.33	3.00	3.17	2.48	0.15	2.8	2.31	3.6	*0.03*
Enjoyment	2.50	3.10	0.16	3.78	2.00	2.17	3.00	3.83	2.91	0.30	3.8	2.67	3.9	0.15
Subscores														
Severity score	1.62	1.76	0.49	1.38	2.26	1.70	3.41	1.53	1.55	0.05	2.18	1.59	1.41	*0.03*
Interference score	2.34	2.73	0.30	3.44	1.83	2.64	3.06	3.33	2.67	0.23	3.18	2.62	3.3	0.13

Means and *p* values for the MDASI individual items and subscores are given broken down by legal sex, race, and ethnicity. *p* values were calculated using *t* tests for legal sex and ANOVA for race and ethnicity. We ran one test per category and per MDASI item. Values less than 0.05 are set in italics

Among race and ethnicity groups, some significant differences in symptom burden were seen (Table 4). People who identified as Latino ethnicity had higher pain burden in both the overall population (mean 2.9 vs mean 1.5) and the subgroup with gliomas (mean 3.2 vs mean 1.5) (Fig. 1B). People who identified as Latino ethnicity also had higher numbness/tingling symptom burden in both the overall population (mean 2.5 vs 1.2) and the subgroup with gliomas (mean 3.1 vs 1.0) (Fig. 1D). People who identified as Latino ethnicity also had higher weakness scores in both the overall population (mean 2.9 vs 1.6) and the subgroup with gliomas (mean 3.1 vs 1.5). Similar results were seen for people who identified their race as Latino (Fig. 1C, E). People who identified as Black race reported higher burden of numbness/tingling, weakness, understanding, speaking, and seizures in the entire cohort but not in the subset of people with gliomas. Of these results, the results for numbness had *p* values less than 0.0003, which is the Bonferroni corrected *p* value for the 180 tests in Table 4.

Overall severity scores (*p* = 0.028 for gliomas, ethnicity) were varied among race and ethnic groups, but the ethnicity difference was mostly driven by lower total severity for people with unavailable ethnicity and differences among people of different races were small with a median between 1 and 2 for most groups.

## Discussion

We conducted a retrospective study of reported symptom burden in primary brain tumor patients based on demographic factors. To our knowledge, this is the first report examining whether symptom burden in primary brain tumor patients varies based on sex, age, race, or ethnicity. In our population, pain, weakness, and numbness/tingling varied significantly across racial and ethnic groups, while all other items remained consistent. We observed no significant differences between the symptom burdens of male and female patients. Potential differences in symptom burden are important not only because of the effect on patients’ lived experience but also because symptom burden correlates with survival [6, 15, 16].

This consistency between male and female symptom burden contrasts with differences seen in other clinical outcomes based on sex. Others have looked at specific symptoms based on sex. One study reported higher seizure activity in male glioma patients over female patients [17]. A higher fatigue burden in female patients was seen in a cohort of 65 people with glioblastoma [18]. That study used a scale more specific for fatigue, the fatigue severity scale, which has not been validated in primary brain tumor patients. Whether the difference results between those studies and ours are due to differences in instrument or in population or random variation since both cohorts are small will require further study.

Although the observation that pain and numbness/tingling burden vary with race and ethnicity is new for primary brain tumors, it is consistent with several other observations in the literature of other cancer types. An abundance of recent research suggests that pain reports vary by race and ethnicity for a variety of cancer types. One study found that Black and multiracial colorectal and lung cancer patients had higher pain severity than White patients [19]. Another study found that non-Hispanic Asian patients, across various cancer types, had a lower cancer pain severity than Hispanic and non-Hispanic White patients [20]. Although one might expect numbness/tingling or pain to correlate with tumor location, that was not the case in our cohort and did not explain the differences seen.

Although our data cannot be used to assess causation, the higher levels of reported pain in people who identify as Black or Latino may be related to undertreatment of pain and numbness/tingling in underrepresented minorities. Several studies have shown that pain is consistently undertreated and underestimated in racial and ethnic minority groups, when compared to non-Hispanic White patients, often in healthcare centers with primarily racial and ethnic minority patients [21]. This undertreatment often results in patients not receiving required analgesics—one study found that 65% of minority patients did not receive proper analgesics while only 50% of non-Hispanic White patients did not receive the same [22]. Awareness of potential biases and the risk of undertreatment is the first step towards addressing disparities.

Our study had several strengths. MDASI data was collected prospectively as part of routine care in English, Spanish, or Chinese, making it representative of real-world experience. We thoroughly analyzed a variety of demographic factors and included a wide scope of individuals in age, sex, gender, and ethnicity. Demographic data were based on patient self-reports in the electronic health record, so are more accurate than assigned demographics. Over 40% of the population was not White, allowing for a diversity of backgrounds.

Our study has some weaknesses. Given the sample size of the population, the number of patients in each individual race is small, which could lead to random errors given the number of comparisons made. In addition, further research is required to determine if our observed differences in pain and numbness/tingling are due to confounding variables or differences in treatment or experience based on race, ethnicity, sex, or age. On average, in both our entire group and glioma cohorts, our patients of Latino race or ethnicity were significantly younger than the patients not of Latino origin in the cohort, similar to what has been reported by others [14]. However, age did not modify the relationship between Latino ethnicity and level of numbness or tingling reported. Lastly, there was heterogeneity in the timing of MDASI-BT, as some patients had been followed for months or years before MDASI-BT was implemented in our clinic. Our power to detect the effect of treatment received on differences in symptom burden was limited.

This was a hypothesis generating study, so we did not correct for multiple comparisons. Therefore, given the number of tests performed, false positives are possible. The results of differences in numbness/tingling based on race in the whole group and the glioma subgroup have *p* values at or below the threshold of a Bonferroni corrected alpha of 0.05, suggesting these may be true effects. Other results with a *p* value less than 0.05 but above 0.0003 (the Bonferroni corrected alpha) may be due to random chance. Our study cannot elucidate the mechanism behind the observed differences. Accordingly, reported differences could be due to biases among healthcare providers, differences in symptom management, or differences in lived experience. Lastly, we were not able to account for or detect possible differential response rates based on demographic factors.

Further research is required to confirm our results in larger populations in diverse geographic areas. If they are confirmed, our results may indicate a need for increased attention to disparities for treatment and prevention, particularly of pain and numbness/tingling, in different groups; a need to increase awareness of specific symptoms; or a need to study the validity of patient-reported outcome measures in diverse groups. Our findings highlight the need for symptom-based research that accurately reflects the diversity of the primary brain tumor patient population.

## Data Availability

Original data is available on request to the corresponding author.
